# Efficacy comparison of 7- and 14-day P-CAB based bismuth-containing quadruple regimen with PPI based bismuth-containing quadruple regimen for *Helicobacter pylori* infection: rationale and design of an open-label, multicenter, randomized controlled trial

**DOI:** 10.1186/s12876-023-03100-y

**Published:** 2023-12-21

**Authors:** Chang Kyo Oh, Hyun Lim, Seung In Seo, Sang Pyo Lee, Chang Seok Bang, Woon Geon Shin, Jin Bae Kim, Hyun Joo Jang, Gwang Ho Baik

**Affiliations:** 1 Department of Internal Medicine, Gangnam Sacred Heart Hospital, Hallym University College of Medicine, 1 Singil-ro, Yeoungdeungpo-gu, Seoul, 07441 Korea; 2 Department of Internal Medicine, Hallym University Sacred Heart Hospital, Hallym University College of Medicine, 22 Gwanpyeong-ro 170-gil, Dongan-gu, Anyang, 14068 Korea; 3 Department of Internal Medicine, Kangdong Sacred Heart Hospital, Hallym University College of Medicine, 150 Seongan-ro, Gangdong-gu, Seoul, 05355 Korea; 4 Department of Internal Medicine, Dongtan Sacred Heart Hospital, Hallym University College of Medicine, 7 Keunjaebong-gil, Hwaseong, 18450 Korea; 5 Department of Internal Medicine, Chuncheon Sacred Heart Hospital, Hallym University College of Medicine, 77 Sakju-ro, Chuncheon, 24253 Korea; 6 Institute for Liver and Digestive Diseases, Hallym University, 1 Hallymdaehak-gil, Chuncheon, 24252 Korea

**Keywords:** *Helicobacter pylori*, Potassium-competitive acid blockers, Proton pump inhibitors, Bismuth-containing quadruple therapy

## Abstract

**Background:**

Owing to its strong acid inhibition, potassium-competitive acid blocker (P-CAB) based regimens for *Helicobacter pylori* (*H. pylori*) eradication are expected to offer clinical advantages over proton pump inhibitor (PPI) based regimens. This study aims to compare the efficacy and adverse effects of a 7-day and a 14-day P-CAB-based bismuth-containing quadruple regimen (PC-BMT) with those of a 14-day PPI-based bismuth-containing quadruple regimen (P-BMT) in patients with high clarithromycin resistance.

**Methods:**

This randomized multicenter controlled clinical trial will be performed at five teaching hospitals in Korea. Patients with *H. pylori* infection who are naive to treatment will be randomized into one of three regimens: 7-day or 14-day PC-BMT (tegoprazan 50 mg BID, bismuth subcitrate 300 mg QID, metronidazole 500 mg TID, and tetracycline 500 mg QID) or 14-day P-BMT. The eradication rate, treatment-related adverse events, and drug compliance will be evaluated and compared among the three groups. Antibiotic resistance testing by culture will be conducted during the trial, and these data will be used to interpret the results. A total of 366 patients will be randomized to receive 7-day PC-BMT (n = 122), 14-day PC-BMT (n = 122), or 14-day P-BMT (n = 122). The *H. pylori* eradication rates in the PC-BMT and P-BMT groups will be compared using intention-to-treat and per-protocol analyses.

**Discussion:**

This study will demonstrate that the 7-day or 14-day PC-BMT is well tolerated and achieve similar eradication rates to those of 14-day P-BMT. Additionally, the 7-day PC-BMT will show fewer treatment-related adverse effects and higher drug compliance, owing to its reduced treatment duration.

**Trial registration:**

Korean Clinical Research Information Service registry, KCT0007444. Registered on 28 June 2022, https://cris.nih.go.kr/cris/index/index.do.

## Background

*Helicobacter pylori* (*H. pylori*) infection is one of most common causes of chronic bacterial infections in humans. Although its prevalence is gradually decreasing, approximately half of the global population is still infected with *H. pylori* [1]. *H. pylori* infection can lead to significant gastrointestinal morbidities, including peptic ulcer disease, gastric adenocarcinoma, chronic gastritis, and low-grade gastric mucosa-associated lymphoid tissue (MALT) lymphoma [2–4]. These diseases can be prevented or treated by *H. pylori* eradication; thus, eradication is recommended in order to treat and prevent these diseases [5, 6].

For effective *H. pylori* eradication, multi-antibiotic regimens combined with anti-secretory agents are recommended. The Maastricht VI/Florence Consensus Report and the 2020 revision of the Korea Guideline for *H. pylori* recommend clarithromycin-containing triple regimen as first-line treatment [7, 8]. However, in recent years, the eradication rate of the clarithromycin-containing triple regimen has declined in many countries worldwide due to growing antibiotic resistance, particularly clarithromycin resistance [9–11]. To overcome the failure of eradication, various regimens such as sequential therapy, concomitant therapy, and bismuth-containing quadruple therapy have been used [7, 8]. When used as a first-line therapy, the bismuth-containing quadruple regimen shows an eradication rate of 90–95%, which is significantly higher than that of the clarithromycin-containing triple regimen in the per-protocol analysis [12, 13]. However, the bismuth-containing quadruple regimen requires four times daily administration for 10–14 days, and high adverse events cannot be ignored in clinical practice [8, 13].

The unsuccessful eradication of *H. pylori* is associated with high gastric acidity and antibiotic resistance. The activity of antibiotics increases when gastric acidity is neutralized. Therefore, it is recommended to maintain a gastric pH above 5 to optimize the action of antibiotics for *H. pylori* eradication [14]. However, currently available proton pump inhibitors (PPI) do not typically achieve the required level of acid suppression for the full 24-hour duration to accomplish this target. Potassium-competitive acid blockers (P-CABs) inhibit gastric acid secretion through the reversible and selective inhibition of H+/K+-ATPase [15]. The onset of action of P-CABs is faster than that of PPI. Furthermore, acid suppression by P-CABs has a longer duration, making it a more potent therapy for *H. pylori* eradication. Recent studies have demonstrated that P-CAB-based regimens are superior to PPI-based regimens in achieving eradication [16]. Moreover, P-CAB-based regimens with shorter duration and less complex drug administration have shown acceptable eradication rate [16, 17].

The aim of this study was to compare the efficacy and safety of 7- and 14-day P-CAB based bismuth-containing quadruple regimens with 14-day PPI based bismuth-containing quadruple regimen and to report the antibiotic resistance profile of *H. pylori* in areas of high (> 15%) clarithromycin resistance.

## Methods/Design

### Study design

This is a prospective, open-label, multicenter, randomized controlled trial. Eligible participants with *H. pylori* infection will be randomized into one of three treatment arms: a 7-day P-CAB-based bismuth-containing quadruple regimen (7-day PC-BMT), a 14-day P-CAB-based bismuth-containing quadruple regimen (14-day PC-BMT), or a 14-day PPI-based bismuth-containing quadruple regimen (14-day P-BMT). The PC-BMT regimens will consist of tegoprazan 50 mg BID, bismuth subcitrate 300 mg QID, metronidazole 500 mg TID, and tetracycline 500 mg QID daily, whereas the P-BMT regimen will consist of rabeprazole 20 mg BID, bismuth subcitrate 300 mg QID, metronidazole 500 mg TID, and tetracycline 500 mg QID daily. The eradication rate, treatment-related adverse events, and drug compliance will be investigated and compared among the three regimens. Antibiotic resistance test will be conducted during the trial, and the data will be used to interpret the results.

Participants will be recruited from five Hallym University-affiliated hospitals (Hallym University Sacred Heart Hospital, Kangnam Sacred Heart Hospital, Dongtan Sacred Heart Hospital, Kangdong Sacred Heart Hospital, and Chuncheon Sacred Heart Hospital) during the recruitment period from January 2023 to June 2024. Personal interviews with open-ended questions via questionnaires and self-reporting will be conducted by telephone on day 7 or 14 after enrollment (completion of eradication regimen) to assess drug compliance and treatment-related adverse events. The interview window is two weeks. At their first visit post-regimen (four weeks after completion of the eradication regimen), we will assess eradication success and check for potential treatment-related adverse events and protocol violations.

We had received approval from the MFDS in Korea and the IRB for human research at Hallym University-affiliated Hospital (No. 2021-09-013) before the study was initiated. This study was registered at cris.nih.go.kr in June 2022 (clinical trial registration number KCT0007444). The study protocol adheres to the CONSORT guidelines and Declaration of Helsinki as reflected in a priori endorsement by the institution’s human research committee. Written informed consent will be obtained before endoscopic examination.

### Study participants

Study participants will include individuals aged 18 years or older who have undergone upper gastrointestinal endoscopy within the past 4 weeks and are proven to have *H. pylori* infection either by the rapid urease test (RUT), the histological examination, or the ^13^C-urea breath test (UBT). Participants with any one of the following criteria will be excluded from the study: (1) history of *H. pylori* eradication, (2) history of stomach resection, (3) history of allergy or adverse events related to the test medications, (4) history of using PPI or P-CAB within 2 weeks before randomization, (5) history of using antibiotics or bismuth containing drugs within 4 weeks, (6) breast-feeding or pregnant participants or participants who do not wish to avoid pregnancy during the study, (7) participants who are taking atorvastatin, simvastatin, lovastatin, ritonavir, indinavir, cyclosporin, pimozide, astemizole, terfenadine, human immunodeficiency virus protease inhibitors (nelfinavir, atazanavir), mizolastine, dihydroergotamine, ergotamine, bepridil, or ticagrelor, and (8) participants who have a hematological disease, a central nervous system infection, an infectious mononucleosis, glucose-galactose malabsorption, Lapp lactase deficiency, galactose intolerance, or Torsades de pointes.

### Outcome measurement

The primary endpoint of this study is the *H. pylori* eradication rate, which will be assessed using intention-to-treat (ITT) and per-protocol (PP) analyses. All the randomized patients will be included in the ITT analysis. Patients who do not visit for follow-up of ^13^C-UBT will be considered treatment failures. Patients who failed to take at least 80% of their allocated drugs or who were lost to follow up will be excluded from the PP analysis. The secondary endpoint will be the frequency of treatment-related adverse events and drug compliance. Drug compliance will be checked at the first visit by independent staff and be determined via pill counts, with good compliance defined as consumption of at least 80% of the total dosage.

*H. pylori* infection will be diagnosed using one or more of the following methods: the RUT, the histological examination, or the ^13^C-UBT. Two biopsy specimens from the gastric corpus and antrum will be taken during an endoscopy for RUT (Pronto Dry New; Medical Instruments Corp., Herford, Germany) or histological examination using Giemsa staining. A UBT (UBiT-IR 300; Otsuka Pharmaceutical Co., Ltd., Tokyo, Japan) measuring the exhalation of ^13^CO_2_ before and 30 min after the ingestion of 75 mg of ^13^C-marked urea will be conducted. UBT after *H. pylori* eradication will be conducted at least 4 weeks after the completion of eradication therapy. Delta over baseline > 2.5‰ will be considered positive. Participants who are taking medications that may impact the UBT findings (e.g., histamine 2 receptor antagonists, PPIs, P-CABs, and antibiotics) will be tested for at least 4 weeks after discontinuation of these medications (the drug-free period is at least 4 weeks).

All participants will be informed about potential treatment-related adverse events, and they will be asked about treatment-related adverse events during the telephone interview on day 7 or 14 after enrollment (completion of eradication regimen). They will also be asked to submit self-report questionnaires at the first visit, which will occur five or six weeks after enrollment, for UBT checking. The severity of the adverse events will be classified into three levels: mild (transient and well-tolerated), moderate (causing discomfort and partially interfering with daily activities), or severe (causing considerable interference with daily activities) [18]. Information about treatment-related adverse events will also be collected through interviews conducted by the doctors and independent staff at the first visit.

### Antibiotic resistance test

The antibiotic resistance test will be carried out by using a methodology previously reported in our previous studies [19]. For the participants who will prospectively conduct endoscopic examination with the RUT or a histological examination to determine *H. pylori* infection, two biopsy specimens sourced from the gastric corpus and antrum will be cultured using an agar dilution test to evaluate antibiotic resistance. For participants who have already undergone endoscopic examination or who have been diagnosed with *H. pylori* infection using UBT, antibiotic resistance tests will be omitted. The biopsy specimen will be cultured and maintained on brucella broth agar supplemented with 5% sheep blood and containing trimethoprim (5 mg/mL), vancomycin (10 mg/mL), polymyxin B (2.5 IU), and amphotericin B (5 mg/mL) under microaerophilic conditions (5% O_2_, 10% CO_2_, 85%N_2_) at 37 °C for 3 to 7 days. All obtained isolates will be stored in brucella broth supplemented with 15% glycerol at 70 °C. A culture will be deemed positive if it contains one or more colonies that are gram negative and positive for oxidase, urease, and catalase, with a spiral/curved rod morphology. For each participant, a single isolate will be collected, and all stored isolates will be thawed and passaged for the agar dilution test. Thawed isolates will be inoculated onto Mueller-Hinton agar supplemented with 5% defibrinated sheep blood for 48 h. The bacterial suspension adjusted to 1 × 10^7^ colony-forming units (equivalent to that of a No.2 McFarland opacity standard by spectrophotometry) will be inoculated directly onto an antibiotic-containing agar dilution plates at various concentrations. After 72 h of microaerophilic incubation, the minimal inhibitory concentrations (MIC) of metronidazole, tetracycline, amoxicillin, and clarithromycin will be assessed using the 2-fold agar-dilution method. Three susceptibility tests will be performed for each sample. The breakpoint of MICs will be determined based on the National Committee for Clinical Laboratory Standards criteria (MIC value > 0.5 mg/mL (amoxicillin), > 1 mg/mL (clarithromycin), > 8 mg/mL (metronidazole), and > 4 mg/mL (tetracycline), respectively [20].

### Calculating the number of clinical trial participants and randomization

In a recent study conducted in Korea, the eradication rate of 14-day P-BMT was reported to be 82.8% in the ITT analysis when used as a first-line therapy [19]. To date, there are no clinical data on the eradication rate of PC-BMT. However, the eradication rate of 7-day and 14-day PC-BMT is expected to be non-inferior to that of 14-day P-BMT. Based on these study results, the eradication rate of PBMT was applied at 82.8%, and the eradication rate of PC-BMT was 89.6% (upper confidence limit of the previous study eradication rate of 82.8%), non-inferiority limit of −8%, and 2.5% significance level (one-sided), with a power of 85%, 97 patients are needed each in the 7-day PC-BMT, 14-day PC-BMT, and PBMT groups. Taking into account a 20% dropout rate, 122 people will be registered in each administration group for a total of 366 people.

Eligible participants will be approached during routine gastroenterology unit visits by the research team for recruitment. A computer-generated permuted random block with sizes of 2, 4, or 6 will be utilized for the randomization process via a web-based system. The randomization sequence will be strictly concealed from the study investigators.

### Statistical analyses

**Fig. 1:**
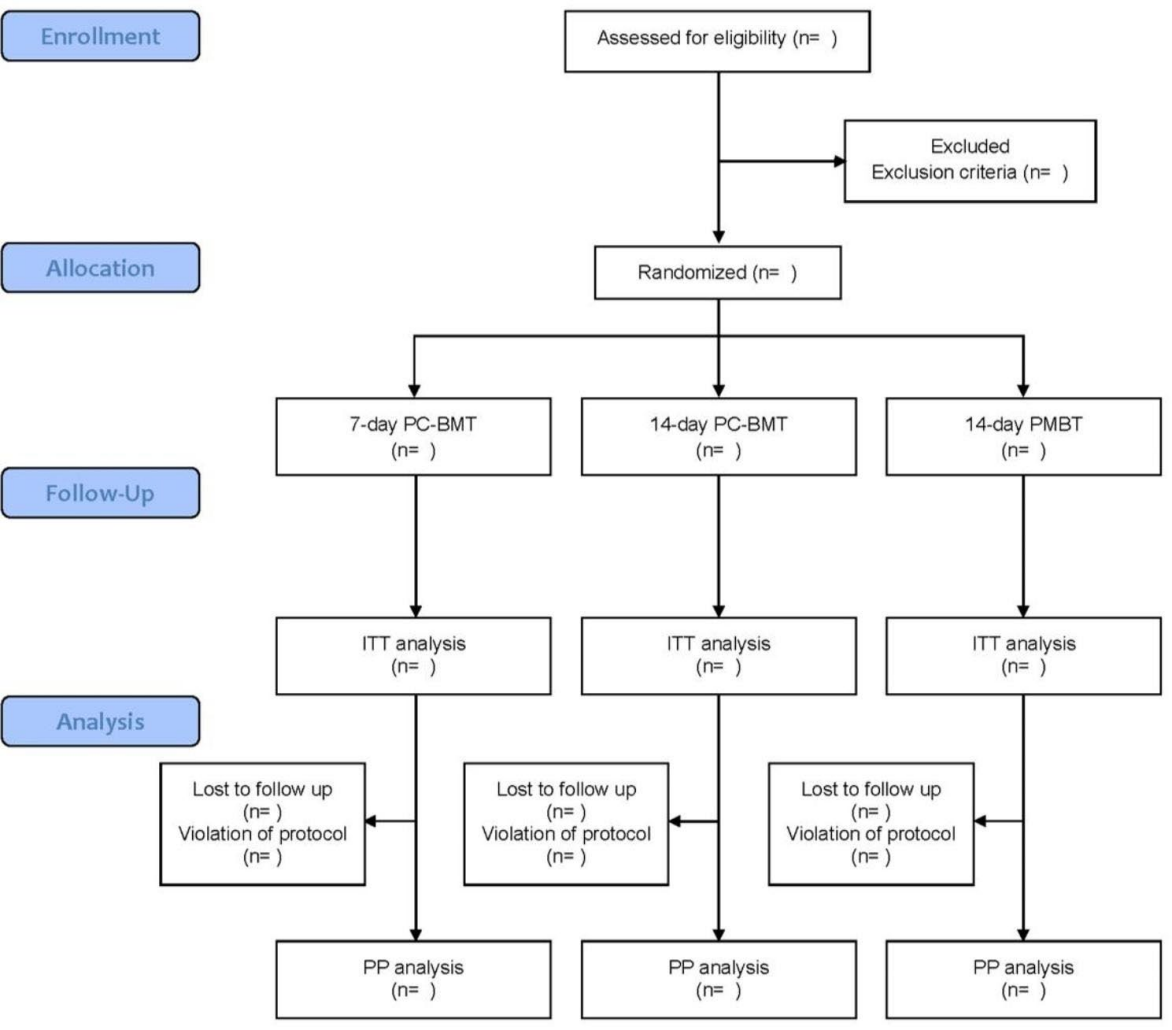
Flow diagram of trial. *PC-BMT* P-CAB-based bismuth-containing quadruple regimen, *PMBT* PPI-based bismuth-containing quadruple regimen, *ITT* intention-to-treat, *PP* per-protocol

In the intention-to-treat (ITT) analysis, all participants who receive at least the first dose of prescribed medications will be included, regardless of their adherence to the medication regimen. For the per-protocol (PP) analysis, only those participants who adhere to the prescribed medication regimen without violating the regulations (where violation is defined as medication compliance less than 80%) will be included and assessed (Fig. 1).

The chi-square test or Fisher’s exact test will be used to test for associations among various categorical variables, and the independent samples t-test will be used for noncategorical variables. Statistical analyses will be performed using SPSS software (version 26.0; SPSS, Chicago, IL, USA), and *p* < 0.05 will be considered statistically significant.

## Discussion

PPI-based regimens are the most common first-line and salvage therapies for *H. pylori* eradication. For first-line therapy for *H. pylori* eradication to be acceptable, the eradication rate should be exceed 80% in ITT analysis and 90% in PP analysis [21]. However, the eradication rate of PPI based regimens has declined. Antibiotic resistance rates, especially resistance to clarithromycin or dual resistance to clarithromycin and metronidazole, are a major cause of the global failure of H. pylori eradication [9–11]. A Korean nationwide study for antibiotic resistance showed that resistance rates against clarithromycin and metronidazole were 17.8% and 29.5%, and multidrug resistance (MDR) rate was 25.2% among quinolone, clarithromycin, metronidazole, amoxicillin, and tetracycline [22]. If the clarithromycin resistance rate exceeds 15% in regions, consensus and guidelines recommend abandoning clarithromycin-containing triple regimen [7]. Preferably, non-bismuth quadruple regimen, sequential or concomitant regimen, or bismuth-containing quadruple regimens are recommended as the first-line therapy. A successful eradication of H. pylori requires a combination of potent acid suppression and effective antibiotics; however, the optimal regimen and duration for *H. pylori* eradication as first-line therapy are yet to be determined, especially in regions of high clarithromycin resistance.

Tailored therapy based on antibiotic resistance test is highly effective and useful to limit the increase in antibiotic resistance by avoiding the use of unnecessary antibiotics [23]. However, antibiotic resistance test is not fully available in clinical practice, and culturing of *H. pylori* is challenging with less than 80% of culture success. Therefore, clinicians should consider the prevalence of antibiotic resistance in the population being treated and current local success rates of specific regimens. Bismuth-containing quadruple regimen is an effective therapy for *H. pylori* eradication, with consistent > 90% eradication rates [8, 12]. Bismuth salt, known for its antibacterial activity, is one of the longest-used drugs. Although the mechanism by which bismuth salt exerts its antibacterial effect on *H. pylori* is not fully understood, evidence suggests that it inhibits the growth of *H. pylori* and has synergistic effects with antibiotics. Moreover, to date, no *H. pylori* resistance to bismuth salt has been reported [24]. It was shown that clarithromycin and metronidazole-resistant *H. pylori* strains become susceptible if administered together with bismuth salt [24]. Therefore, bismuth-containing quadruple regimen is recommended both by the Maastricht IV/Florence Consensus Report and by the Second Asia-Pacific Consensus Guidelines as an alternative first-choice therapy to clarithromycin-containing triple regimen in areas with low clarithromycin resistance (< 15%) [7, 25]. However, in regions with a high prevalence of clarithromycin resistance (> 15%), it is recommended as the first-line therapeutic option [7].

Patient adherence to prescribed medications and adverse effects are also important factors for successful *H. pylori* eradication. However, long treatment duration and complex drug administration may reduce drug compliance and increase adverse events. Bismuth-containing quadruple regimens are typically administered four times daily for a treatment duration of 10–14 days [7, 8]. Several studies have reported that all treatment-related adverse effects of bismuth-containing quadruple regimens are mild and similar to those clarithromycin-containing triple regimens. However, a recent meta-analysis has found that the frequency of adverse events is significantly higher in patients receiving bismuth-containing quadruple regimen than in those receiving other treatment regimens, with a pooled relative risk for adverse events of 1.64 (95% CI, 1.11 ~ 2.44; P = 0.01) [8]. This higher incidence of adverse events could be the main reason for poor compliance, leading to treatment failure and subsequent development of resistant bacterial strains.

Gastric pH is an important factor affecting *H. pylori* eradication. Owing to their strong acid inhibition, P-CAB-based regimens are expected to offer clinical advantages over PPI based regimens. Furthermore, because P-CAB is acid-stable and is not dependent on the CYP2C19 genotype or activation of parietal cells compared to PPI, P-CAB in *H. pylori* eradication regimens have obtained considerable interest [26]. Several recent meta-analyses, although mostly based on retrospective studies with low levels of evidence, have shown the superiority of P-CAB based regimens over PPI based regimens in terms of efficacy, adverse events, and drug compliance [16, 27]. This has led to the suggestion that the more potent acid suppression by P-CAB may enable shorter durations of *H. pylori* treatment and may even overcome antibiotics resistance [16, 27]. A previous meta-analysis has shown that PPI based bismuth-containing quadruple regimen for 7 days is less effective than when given for 10–14 days [28]. Therefore, in consensus and guidelines, the recommended PPI based bismuth-containing quadruple regimen is 10–14 days. However, more potent acid suppression by P-CAB could allow shorter durations of bismuth-containing quadruple regimens, which would decrease adverse events and increase drug compliance.

This study has several strengths. First, it is a prospective, open-label, multicenter, randomized controlled trial that will demonstrate the efficacy and safety of 7- and 14-day PC-BMT compared to 14-day P-BMT in areas of high (> 15%) clarithromycin resistance. Doctors and patients will be aware of the prescribed drugs because of the differences in the administration method and duration for each arm. However, this trial will only consider the success rate of eradication, treatment-related adverse events, and drug compliance as outcomes. Therefore, it is believed that an open-label design will not have any impact on the procedure or results of the trial. Second, the availability of antibiotic susceptibility data is another major strength of this study. These data will be used to interpret the results and assess their impact on eradication rates.

However, this study has several limitations. First, despite significant efforts to minimize missing data in antibiotic resistance test, it is acknowledged that some data may still be missing. Second, our study results may have limited applicability in countries where bismuth preparations are unavailable. Third, although PBMT and PC-BMT will show a high eradication rate, some patients may fail to eradicate, and we do not provide information on rescue therapy.

In conclusion, our study will demonstrate that the 7-day or 14-day PC-BMT is well tolerated and achieve eradication rates similar to those of 14-day P-BMT. Furthermore, the 7-day PC-BMT will show fewer treatment-related adverse effects and higher drug compliance, owing to its reduced treatment duration. Therefore, 7-day PC-BMT may be a first-line treatment option for *H. pylori* with the advantage of having a shorter duration, especially in areas of high clarithromycin resistance.

## Data Availability

The datasets used and/or analysed during the current study are available from the corresponding author on reasonable request.

## List of abbreviations

P-CAB: potassium-competitive acid blocker
PPI: proton pump inhibitor
PC-BMT: P-CAB-based bismuth-containing quadruple regimen
*H. pylori*: *Helicobacter pylori*
MALT: mucosa-associated lymphoid tissue
BID: twice a day
TID: three times a day
QID: four times a day
RUT: rapid urease test
CUT: C-urea breath test
ITT: intention-to-treat
PP: per protocol
MIC: minimal inhibitory concentration
MDR: multidrug resistance
